# Evolution of a community-centred experiential learning module: A mixed-methods approach to promote social accountability and community partnership in undergraduate medical education

**DOI:** 10.15694/mep.2020.000217.2

**Published:** 2021-07-19

**Authors:** Yuchen Gao, Ulrich Teucher, Erin Wolfson, Krista Baerg, Nicole Graham, Sandra Pfeifer, Krista Trinder, Megan Wells, Juan Nicolás Peña-Sánchez

**Affiliations:** 1 Department of Sociology; 2 Department of Sociology; 3 Department of Psychology; 4 Department of Psychology; 5 Division of Social Accountability; 6 Division of Social Accountability; 7 Department of Pediatrics; 8 Department of Pediatrics; 9 Inclusion Saskatchewan; 10 Rainbow Youth Centre; 11 Undergraduate Medical Education; 12 Undergraduate Medical Education; 13 Department of Community Health & Epidemiology; 14 Department of Community Health & Epidemiology

**Keywords:** social determinants of health, community-based education, service learning, undergraduate medical education, community engagement

## Abstract

This article was migrated. The article was marked as recommended.

**Background:** Education in social determinants of health (SDH) has become an important part of medical curricula, facilitated increasingly through students’ experiential learning with communities. The Community and Workplace Centred Learning Experience (CWCLE) module of the University of Saskatchewan, Canada, intends to integrate and extend second-year medical students’ attitudes, skills, and knowledge about SDH and community resources. We aimed to: 1) Solicit students’ self-evaluation of their ability to perform module learning objectives, 2) Assess module impact on student attitudes toward SDH, 3) Obtain feedback from community partners and students about their community experiences, and 4) Use feedback to collaboratively develop recommendations to enhance the CWCLE module.

**Methods:** We used a mixed-method approach to combine quantitative data with stories and personal experiences. We developed an online survey for two cohorts of students who had completed the module, soliciting students to self-evaluate their perceived abilities to perform the module’s learning objectives and evaluating students’ attitudes towards SDH. We invited representatives from community agencies involved in the CWCLE module to participate in focus groups. We also held separate focus groups with students who participated in the online survey to elaborate on their survey comments.

**Results:** In total, 145 students participated in the online survey (response rate=72.5%). Eleven community agency representatives and seven students participated in five focus groups. Our results demonstrate that medical students benefit from community-based experiential learning of SDH and community resources. We trace evaluations and discussions in the ongoing development of this community-based experiential learning module from its initial, primarily medical-school driven designs, towards a substantial involvement of community-based organizations in its operation and continuing redevelopment.

**Conclusions:** Our mixed method offered us a better understanding of module impact and opportunities for improvement. This module evaluation and reform generated opportunities for community partners to influence decisions in medical education and led to a collaborative evolution of a community-centred learning experience. Medical schools should actively engage community partners in teaching behavioural and social components of the curriculum and acknowledge their partners’ expertise to promote community engagement and social accountability in medical education.

## Introduction

Education in social determinants of health (SDH–the conditions, forces, and systems that influence people’s health) is recognized as an important component in medical curricula (Karen *et al.,* 2019). This training involves fostering, in medical students, a sense of social accountability to address social inequities, particularly among disadvantaged communities ( Yeo, 2017). The WHO (2020) emphasizes the implications of health disparities caused by SDH and medical schools’ obligation to direct their activities towards priority community health concerns ( Boelen and Heck, 1995). Since it is difficult to teach social aspects of medicine in traditional classroom settings ( Meili, Fuller, and Lydiate, 2011), medical education is often complemented by experiential learning in the communities, requiring partnerships between Colleges and communities. One important form of this learning is Community-Based Education (CBE; Doobay-Persaud *et al.,* 2019).

CBE that involves learning activities carried out in community settings is becoming an essential component in medical curricula ( Magzoub and Schmidt, 2000), and can take different forms at pre-clerkship level: service project, community-based participatory research, and/or neighbourhood visits ( Choulagai, 2019; Doobay-Persaud *et al.,* 2019). CBE can include short-term initiatives, whether as singular program ( Duffy *et al.,* 2014) or course component ( Asgary *et al.,* 2016). Other initiatives seek longitudinal community collaborations, for example, as four-year nonclinical, urban population health program ( Girotti *et al.,* 2015), or as longitudinal policy and advocacy service project ( Bakshi *et al.,* 2015). One essential CBE component utilizes reflection, as both instructional strategy ( Bullock, Jackson, and Lee, 2014; Meurer *et al.,* 2011) and assessment ( Bernstein, Ruffalo, and Bower, 2016). CBE program-level assessments commonly take the form of student surveys, including self-assessment of knowledge, skills and attitudes, affective assessment of program elements ( Doobay-Persaud *et al.,* 2019).

Introduced in the 1990s ( Seifer, 1998), service learning has become CBE’s most common form ( Doobay-Persaud *et al.,* 2019), providing “a structured learning experience that combines community service with explicit learning objectives, preparation, and reflection” (p. 273). It has been effective in teaching social accountability and in encouraging work in underserved areas ( Meili, Fuller, and Lydiate, 2011). As community agency representatives actively participate in CBE’s service-oriented programs and contribute to teaching medical students, medical schools and community agencies should all benefit ( Choulagai, 2019). However, community educators’ contributions to advising and facilitation are not well acknowledged nor compensated ( Doobay-Persaud *et al.,* 2019). Little research examines medical students’ and community partners’ experiences with, and opinions about, CBE. For example, Haq *et al* (2013) asked community leaders to comment on students’ performances, and on the formers’ capacity to accommodate student involvement; DeHaven (2011) surveyed community partners’ attitudes towards the students and programs they were involved with; and other researchers identified needs, to evaluate community-defined outcomes ( O’Brien *et al.,* 2014), to recognize and acknowledge community agencies’ supports, and to engage the latters’ expertise in CBE development, implementation, and evaluation ( Choulagai, 2019). The goal of CBE is for students to learn medicine not only in the community but also from the community ( Choulagai, 2019).

At the University of Saskatchewan’s College of Medicine, its undergraduate medical curriculum has four “Medicine and Society” courses with multiple experiential learning modules. One of these modules, the CWCLE (Community and Workplace Centred Learning Experience) provides 14 hours of experiential learning for second-year medical students to integrate and extend attitudes, skills, and knowledge about SDH, and learn about community resources. Since our Medical College offers two main training sites for its medical students in the cities of Regina and Saskatoon, we collaborated with local community agencies (i.e., non-profit community-based organizations or community-based programs) in both cities to offer this module to our students. Feedback received from students and community agencies involved in this module suggested needs for improvement. Therefore, we aimed to: 1) Solicit students’ self-evaluation of their ability to perform module learning objectives, 2) Assess module impact on student attitudes toward SDH, 3) Obtain feedback from community partners and students about their community experiences, and 4) Use feedback to collaboratively develop recommendations to enhance the CWCLE module. We expected that the results of our study would lead to making incremental changes to the module, but we did not expect that we would be addressing a more fundamental issue, which is how the university communicates and collaborates with its community partners regarding the training of medical students.

## Methods

**Figure 1.**
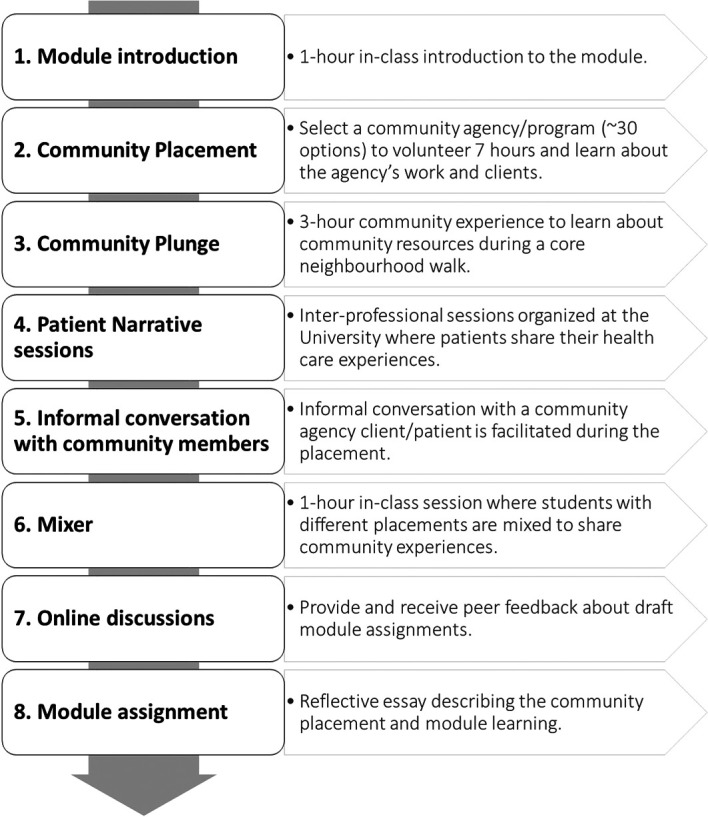
Original Module Design promoted by Medical College. Source: the authors.

At the beginning of our study, the CWCLE module had six learning objectives ( Table 1) and eight activities that span the program’s two terms in second year ( Figure 1).

**Table 1.** Self-evaluated ability to perform learning objectives before and after completing CWCLE.

Module learning objective	Before		After		p-value
	Mean (SD)	Median (IQR)	Mean (SD)	Median (IQR)	
1. Explain how selected community agency or workplace addresses the SDH of its clients, employees and/or volunteers	3.14 (1.07)	3 (2)	4.17 (0.76)	4 (1)	
2. Identify how socio-political context affects the work of community agencies in addressing SDH	3.24 (1.05)	3 (1.75)	4.09 (0.75)	4 (1)	
3. Explain the role of work, working conditions, and occupational health and safety policies on health and well-being of employees/volunteers at the agency or workplace selected	3.11 (1.05)	3 (2)	3.99 (0.76)	4 (0)	
4. Explain the roles physicians can play in working with community agencies and workplaces to enhance health and well-being	3.25 (1.01)	3 (1)	4.07 (0.72)	4 (1)	
5. Promote relationships with community agencies or workplaces selected to collaborate with and advocate for initiatives addressing SDH	3.15 (1.09)	3 (2)	4.01 (0.73)	4 (0)	
6. Recognize examples and non-examples of patient- and family- centred care	3.56 (1.02)	4 (1)	4.18 (0.72)	4 (1)	

### Mixed methods approach

To evaluate the CWCLE experiential learning module, we devised a mixed-methods data-analytic approach, for utilitarian reasons: to combine statistical measures (quantitative data) with stories and personal experiences (qualitative data). We selected this mixed approach, convinced that “this collective strength provides a better understanding of the research problem than either form of data alone” ( Creswell, 2014, p. 2). The University of Saskatchewan Research Ethics Board provided an exemption from ethics review based on article 2.5 of the Tri-Council Policy Statement. Interviewed participants were assigned fictitious names and they provided written consent for quotes attributed to them to be released only under their assigned fictitious names.

### Quantitative component

We developed an online survey for two cohorts of students who had completed the module at the end of the 2016-17 and 2017-18 academic years. All students in these two cohorts were invited to participate anonymously through One45 (software used by the College to manage the medical program).

The survey included a self-assessment, evaluating students’ perceived abilities to perform each of the module’s learning objectives, both after the module and retrospectively to before the module, on a Likert scale from ‘Not at all’ (1) to ‘Very much’ (5). Items to measure attitudes towards SDH were derived from a questionnaire previously developed ( Fuller *et al.,* 2016; Lemstra, Neudorf, and Beaudin, 2007) with permission from the senior co-author of the questionnaire, Cory Neudorf. One item asked participants “Would you say that people with low income or low education are much less likely, less likely, equally likely, more likely, or much more likely to suffer from poor health than people with a high income?”, on a scale from 1 (much less likely) to 5 (much more likely). Another five items explored participants’ beliefs whether unhealthy eating, lack of physical activity, illegal drug use, alcohol abuse, and smoking are individual choices or associated with low income and educational level; these items were rated on a scale from 1 (individual choice) to 5 (associated with income and education). Students’ satisfaction with the module components was also measured on a scale from 1 (very dissatisfied) to 5 (very satisfied); see Supplementary File 1 for a questionnaire sample.

Means, standard deviations (SD), medians, and interquartile ranges (IQR) were reported. Wilcoxon Matched-Pairs Signed Rank tests were used to compare differences in students’ perceived abilities to perform learning objectives and attitudes towards SDH before and after module completion. Friedman’s ANOVA test was used to determine rank differences in students’ satisfaction across module components, with posthoc analysis using Wilcoxon Matched-Pairs Signed Rank Tests. Analyses were completed using SPSS® software (version 24) and p-values below 0.05 were considered statistically significant.

This quantitative component informed us in general ways about students’ own evaluations of whether what they knew about SDH had changed during the course of the module, including their placements with community agencies, and what they thought about the usefulness of the module’s components and assessments. Notwithstanding, we also wanted to learn details about students’ and community agency representatives’ experiences regarding learning and teaching students about SDH during the CWCLE module. For this, we needed qualitative interview data to be gathered in focus groups with students and, separately, with community agency representatives.

### Qualitative component

We invited representatives from community agencies that were involved in the CWCLE module to participate in focus groups, with the purpose of evaluating and revising the module to review and improve medical students’ learning with community agencies. Twelve agency representatives (out of 26) accepted our invitation. Eight representatives were from community agencies in Saskatoon and four from Regina; the majority (n=11) were women. The representatives were divided into three focus groups, guided by two interviewers. Supplementary File 2 has a copy of the interview guide used in the focus groups with community agency representatives. Due to scheduling conflicts, one representative was interviewed individually. We also held two separate focus groups with students who had participated in the online survey (seven out of 145) to elaborate on their survey comments. Three students were from the 2016-17 academic year and four from 2017-18; most student participants were males (n=4). See in Supplementary File 3 a copy of the interview guide used in the focus groups with medical students.

Interview guides (Supplementary Files 2 and 3) included questions for general feedback; module activities; time management; students’ achievement of learning objectives; and communication among students, community agencies, and the College of Medicine. Much of the discussions in the focus groups centred on the Plunge, student placements, and the relationship and communications between community agencies and the College of Medicine, and the College and the placed students. Interviews lasted between 30 minutes (individual interview) and one hour (focus groups).

Following transcription, the interviews were systematically coded and analyzed for themes, following a common six-step process outlined by Braun and Clarke (2006). This process begins with (1) repeated close readings of the collected data (e.g., transcripts), scrutinizing for possible points of interest, their potential meanings and patterns, and taking note of these first points; (2) systematically identifying and categorizing such points throughout the data set as “codes”; (3) reducing or expanding, and selecting from or grouping, these initial codes into more central, potential “themes”; (4) determining consistency throughout the whole data set between these “themes” and the evidence provided by the codes, and establishing a first overview or map of these themes for the data set; (5) determining consistency between the map of themes and the overall analysis that is being attempted, by reducing or expanding, and (re)organizing and (re)entitling the themes; and, finally, (6) situating and editing the analysis for consistency between initial research question and literature review as well as the conclusion. The more systematically these steps are followed, the better the quality of a qualitative analysis will be.

In this study, two researchers (the first and second author) individually read, closely reread, coded, and came up with themes and suggestions for an overall analysis from all focus group manuscripts transcripts. The team leader (the corresponding author) was included in the detailed discussions of the pros and cons of selecting some themes over other themes. The first author coded and analyzed for themes that emerged from the medical student focus group data and wrote up a draft analysis, while the second author coded and analyzed for themes from the community agency representatives’ data and wrote up his draft analysis. This process helped to sharpen possible similarities and differences between agency members and students, and clarify the identification of themes of mutual and variant interest between the two constituent groups of participants over the course of further meetings. We aimed for a representative selection of themes, in terms of breadth and depth of materials discussed in the groups and the single interview. In discussions that included the other research team members, we achieved consensus on our analysis and began to integrate the quantitative with the qualitative data analysis to render the manuscript consistent and coherent.

### Collaborative Working Group to enhance the module

A Working Group was formed to evaluate the analyses and formulate recommendations for improving the CWCLE module. This ad hoc Working Group included a medical student representative, four community agency representatives, and four university researchers who had led the data analyses. Following a discussion of the analyses, the Working Group collaboratively highlighted module strengths, identified opportunities for improvement, and agreed on a list of recommendations for restructuring the module. During the 2018-19 and 2019-20 years, a multistage process was developed to consider and implement the formulated recommendations.

## Results/Analysis

### Students’ assessment of learning outcomes and module components

In total, 145 medical students participated in the online survey (response rate=72.5%). The students reported an enhanced ability to perform the module learning objectives after completing the CWCLE module, p-values

When comparing students’ attitudes towards SDH before and after the module, students tended to consider somewhat more (following the module) that people with low income and education are more likely to suffer from poor health (Z=-4.78, p

The mean of students’ satisfaction with the CWCLE module as a whole was 3.09 (SD=0.96), with a median of 3 (IQR=1). Significant differences across module components were identified, χ2(3)=55.18, p

**Table 2.** Students’ satisfaction with the module components (n=145).

Component	Mean (SD)	Median (IQR)
Community placements	3.78 (1.09)	4 (2)
Informal conversation with a client/patient	3.32 (1.14)	4 (1)
Patient Narrative sessions	3.08 (1.09)	3 (2)
Community Plunge	2.90 (1.39)	3 (2.5)
In-class introduction	2.78 (1.02)	3 (1.5)
Module reflective assignment	2.54 (1.08)	3 (1)
In-class mixer	2.47 (1.09)	2 (1)
Online discussions	2.36 (1.13)	2 (2)

### In-depth understanding of learning and working experiences with the CWCLE module

In this section, we address the main themes identified in students’ and agency members’ discussions. These themes included: the values of experiential learning; the pros and cons of the Plunge; experiences with placements; and relative presence or lack of reciprocity in the partnership between agencies and the College of Medicine. The first three themes built on the feedback by both community agency members and students; the fourth, final, theme (“reciprocity”), was impressed on us by community agency representatives.

### The value of the module – experiential learning

In the focus group discussions, we identified “experiential learning” as one of the central themes. According to community agency representatives, the goal is for students to “interact with our newcomer clients or interview them and learn about their [SDH]” (Vicky) so that “they really, empathetically, understand what’s going on for people” (Dolores). The value of experiential learning was also acknowledged by students, as stated by Amanda: “...how can we serve people if we don’t understand where they’re coming from...having this first-hand exposure is extremely valuable for us in the curriculum”. This theme of experiential learning signifies, as agreed to by all focus group participants, that community-learning provides opportunities for students to observe first-hand, rather than read in textbooks about, the conditions of the underserved communities and the conditions that their future patients live in. Thereby, students learn social accountability and, in partnership with communities, come to understand how different organizations provide services to those who are affected by SDH. There was close agreement between community agencies and medical students on the positive value of this experiential learning module.

### Community Plunge – can it be improved?

There was considerable feedback from both medical students and community agencies about the neighbourhood walk of the Plunge, revealing strong divisions between and within the groups. Although participants felt the format might be inappropriate or disrespectful, they considered it to be a promising component of experiential learning. For example, students preferred exploring the community service locations by a walk, rather than listening to an in-class presentation. This value of the walk was well explained by Amanda:

... another positive with the walking...and actually going to the place instead of having a PowerPoint is [that] I realize some of the barriers that were there for someone who may have a disability, for someone who might have kids, for someone who doesn’t even practice getting dressed in the morning, for someone who has severe mental illness to even get to these places to get the support is a challenge.

Other students acknowledged an initial fear or anxiety before the Plunge but saw value in the event after completing it. The Plunge participants also appreciated their community-savvy tour guides’ knowledgeability in matters of community and available resources. In addition, students mentioned the potential benefit in having the Plunge at the module opening to familiarize themselves with community agencies and programs before selecting a placement. Community agency representatives, too, appreciated the neighbourhood walk, perhaps best captured by Jennifer (“awesome idea”; “students were blown away by the number of diverse services”). However, students and community agency members also had concerns about the “walking around” format of the Plunge, which led to very strong, negative feelings towards the Plunge, finding it “uncomfortable”, “disrespectful”, “embarrassing”, akin to “tourists visiting a zoo”, or “window shopping poverty”. According to one student: “I felt like we are kind of putting people in fish bowls and we’re kind of just staring at it from this outside perspective” (John). Community agency representatives also cautioned that there was not enough time to connect with community members to hear lived experiences and that the Plunge would provide little more than a glimpse into the actual difficulties of life in the community. Both the community agencies as well as the medical students were similarly divided over the current format of the “Plunge.”

### Placements – immersed in the community or not enough?

The module component that students regarded the most beneficial was the placement. For example, one student considered that “the experiences with the agency that I had were great and I definitely took away way more than I was expecting to” (Michael). Others suggested to further “decrease the amount of in-class time we spend talking about these things and increase the amount of time we’re actually present” (John) so that they could “be doing more participation and be a bit more actively involved” (Michael). Also, students appreciated the variety of placement options to choose from, while those who were unable to select their first choices came to recognize the value of attending an unfamiliar agency. Several community agency members considered the placements a very positive experience (e.g., “I love having students come in”, Darlene). However, some felt that the placement was “too short, needs more time; . . . is that truly helping them to just learn so little about so many things?” (Vicky), leaving students with too little opportunity to understand local backgrounds of poverty and its effects on health. As Dolores explained, “[i]t’s difficult, within seven hours . . . (to) understand what’s going on for people who have lived experiences of living in poverty”. Moreover, Jennifer noted that “people in our community take a long time to build trust with people”. The variation in responses may have to do with agencies where social justice issues are more in the foreground then, say, acute physical health issues. In any case, rather more than less immersion with the communities was wished for by both students and community agency representatives.

When students were asked about the module’s reflective assignment components (i.e. reflective essay, online discussions, and in-class mixer), they felt that the assignment seemed forced and redundant, should be shortened and combined, and any time saved used for more experiential learning in the community. Moreover, students requested more flexibility in choosing their own preferences for reflection and sharing lessons learned with medical students in other years.

### Reciprocity – what is in it for community agencies?

Students and agencies that participated in the focus groups (and the single interview) agreed that the CWCLE module was a worthy pursuit. When asked about the interactions between students, agencies, and College of Medicine, two agency members’ answers captured the general sentiment, “I don’t have any complaints” (Val), and “No problem at all. Everybody has been so nice and accommodating” (Debra). Some agencies, likely those that had students from multiple universities and institutions participating, were finding themselves “getting to the point of fatigue” (Michelle). However, the theme most often discussed, and controversially, among agency members was “reciprocity”, regarding a need for benefits not only for medical students but also for community agencies. Several members appreciated perceived benefits; in the words of one member, the placements hold “mutual benefits for both sides,… [students] talk one-on-one with [a client’s] family which builds appreciation for how the agency supports families and we [agency] benefit from people [students’] understanding what [the agency] is about” (Stacey). Another noted that agency members and their clients can also “learn from students” (Jason) who could share their medical knowledge usefully. Some focus group participants went further, discussing reciprocity on a systems level, as the College, so far, had been retaining the authority for CWCLE module design while shouldering the agencies with the facilitation of students’ placements. One member pointed out that any expertise with community engagement and impact of SDH in the community does reside with the agencies who, in the interest of reciprocity, should be given module design authority and be compensated for their work. In this way, agencies were hoping for a comprehensive partnership not only with the medical students but also with the College. Perhaps because students perceive learning in their community placements as a service, reciprocity did not present itself as an issue to them, unlike for the community agencies who would see their role as contributors to medical education (albeit without remuneration or programming authority).

### Working Group recommendations and module reforms

Following discussions of the evaluations, the ad hoc Working Group recommended to the College to keep the module in the medical curriculum, but to improve students’ community learning experiences and community agency engagement. Main recommendations included: 1) Redesign the Plunge in close collaboration with community agencies; 2) Enable students to make more informed choices regarding placements (e.g., provide more materials from the agencies and/or previous medical students who completed the module); 3) Emphasize reciprocity and other community engagement principles in all module components; 4) Promote student self-directed learning and modify module assessment plan; 5) Increase hours spent in communities.

**Figure 2.**
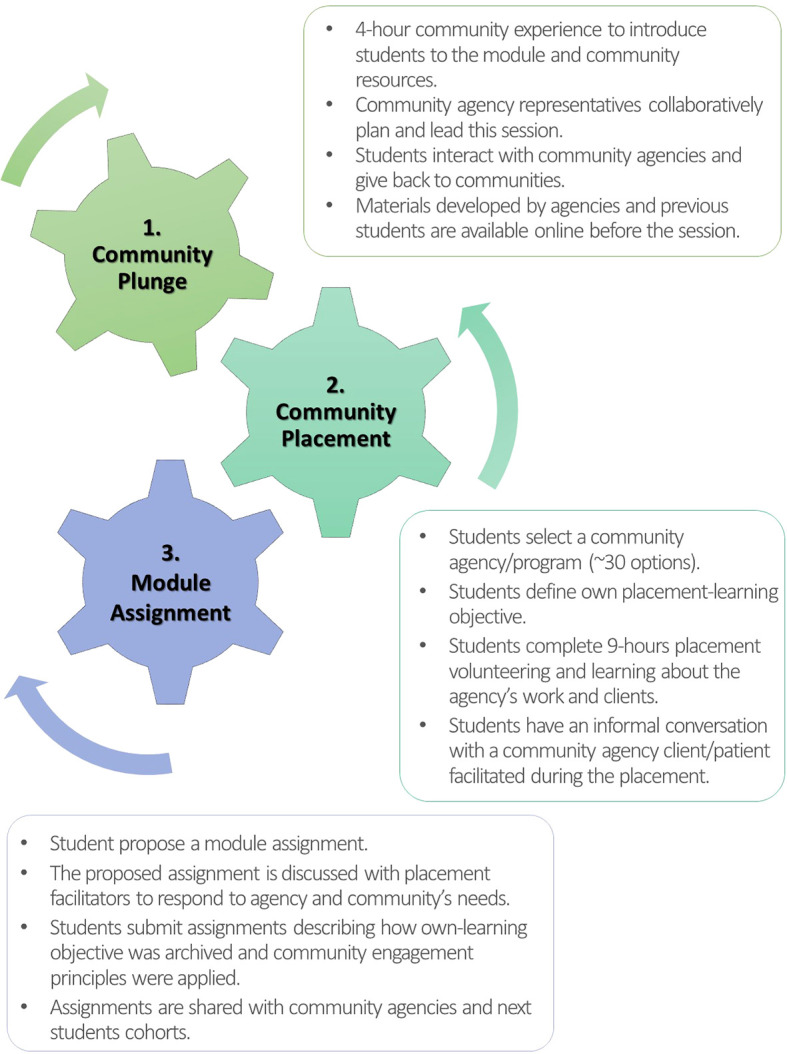
Redesigned Module developed in partnership with community agency representatives. Source: the authors.

Subsequently, the module was redesigned in close collaboration with community agency representatives ( Figure 2). The Plunge was integrated as an introduction to the placements. Rather than a neighbourhood walk, the new format is a four-hour experiential learning activity, featuring structured visits to selected community agencies, as well as so-called Community Learning Café Conversations, a Community Agencies Fair, community members sharing lived experiences, and small group discussions around case scenarios designed by community agencies to address SDH, community needs, and agency services. While versions of the Plunge may vary between the two sites (Regina and Saskatoon), based on local community agency recommendations, the core concept and activities (i.e. module introduction, community agency visits, debriefing session) are equivalent. In both cities, the Plunge begins with Indigenous Elders’ prayers (Elders are individuals with the highest degree of understanding of Indigenous traditions and practices, and the foremost teachers and role models) and the activities are led by community agency representatives. This new version of the Plunge is meant to contextualize the placement experience upfront, enhancing understanding and underscoring the importance of collaboration between students and community members.

The College now also provides students with donation boxes (i.e., boxes filled with items that community agencies need for their clients, such as hygiene products, non-perishable foods, etc.), to give to the agencies during their visits at the Plunge. These donations serve as one form of reciprocity and a way to give back to the community, as was recommended by community agency representatives when co-designing the Plunge. Moreover, Community agencies are now receiving a compensation for the time they dedicate to the module, especially the Plunge. At the current time, a small amount is offered as a token of appreciation for agencies’ commitment and time.

In the redesigned module, students are more engaged in their community learning experiences. Before the Plunge, they can access online materials (e.g. pamphlets, videos, reports, etc.) about the community agencies and programs developed by agencies and previous student cohorts. Following the Plunge, students select their placements, define their placement-learning objectives, and propose and submit module assignments in ways that they find to be more meaningful. The assignments (e.g. posters, infographics, slide presentations, videos, poems, painting, draft surveys, etc.) can be used by community agencies to potentially benefit their members. Lastly, to increase placement hours, three other components of the original module (Patient Narratives, Mixer, and online discussions) were removed, leading to the Plunge, placements, and assignments to be three integrated elements of the new module ( Figure 2).

Since the implementation of the recommendations, community agencies have been generally appreciating their engagement in the redevelopment of the module and the changes put in place, expressing gratitude for “the partnership” and “the opportunity to be involved and the token of gratitude”. Similarly, students generally have been appreciative of the module’s new format as well, in written feedback (e.g., “CWCLE experience is extremely valuable”; “I really enjoyed the CWCLE component of the course”; “enjoyed the Plunge in September and thought it was really well done”; “the Plunge [was] a fantastic use of our time…a great introduction to some of the community resources”). Further recommendations (e.g., formal collaborative agreements between the College and community agencies) are currently under review for implementation in the coming years.

## Discussion

We collected data from two cohorts of medical students (145 participated in an online survey and seven participated in focus groups) and from eleven community agency representatives to evaluate and collaboratively reform the CWCLE module. Students reported an enhanced ability to perform all the module learning objectives after the module, observing a remarkable change on explaining how the community agencies address SDH. Also, the CWCLE had evidence of a positive impact on students’ attitudes towards SDH. Qualitative focus group data provide detailed insights into how and why medical students and community agencies appreciate and benefit from this community-based experiential learning and allow us to recognise opportunities to improve the module and the relationships with community partners. This approach let us move from a medical-school driven design towards a collaborative module that has substantial involvement of community-based organizations in its operation and continuing redevelopment.

We want to highlight that the module evaluation reported here demonstrates some of the many beneficial opportunities that CBE can provide for medical students learning the relevance of SDH, confirming current literature on SDH teaching ( Claramita *et al.,* 2019). The two experiential components of the module, Plunge and placement, offer students first-hand experiences of the significance of SDH and health disparities, and an understanding of community agencies’ commitments to address those issues. The module thereby confirms the value of service-learning in the field and the collaborative teaching of SDH. We think of CBE through a Systems Thinking framework; such frameworks assert that “the effects or outputs of any system are dependent on the interactions of its parts and that studying the parts in isolation will not provide an accurate picture of the system” ( Waldman, 2007, p. 279). From this view, CBE, and especially service learning, bridges the gap of social-and-clinical medicine and facilitates collaboration between medical school and community organizations ( Bullock, Jackson, and Lee, 2014).

Moreover, and perhaps even more importantly, this research reveals the value of engaging and empowering community agencies in the module’s operation and continuing redevelopment. In fact, community agency representatives did not require empowerment; they made clear a need for more reciprocity and recognition from the university. They also drew attention to their practical expertise in the effects of SDO in their communities as a more than complementary and rather essential contribution to the students’ classroom education. Since the Working Group recommendations were put into practice, community agencies have been designing and leading module activities (e.g., Plunge); the College and medical students have put reciprocity into their actions, and the College actively seeks community agency feedback on their working experiences with medical students and the College. The module leaders will continue to identify and nurture what we have collaboratively identified in this study as common interests and goals. As Bullock, Jackson, and Lee (2014) suggest, needs assessments should be conducted to create the foundation upon which students’ learning and services build, to ensure that the program “upholds the mutuality and maintains integrity of community partnerships” (p. 137). In other words, our evaluation of this module yields to a considerable reform in scope, from merely updating students’ learning requirements “in the field” to acknowledging community agencies as essential and co-equal expert partners who need to be consulted in the education of medical students.

Our research could provide curriculum guidance on CBE, identified by Claramita *et al* (2019) as much needed in the CBE literature to enhance undergraduate medical education. Our findings demonstrate that collaborative partnerships between community agencies and Medical Colleges are indeed necessary for modules such as CWCLE to work and to continue growing. Efforts in soliciting feedback from community agencies to promote engagement have so far proven to be successful. While challenges remain in moving forward (e.g. limited curriculum time, limited community and University resources, crowded medical curricula, priority given to clinical experiences, etc.), this is also a time for opportunities. Both medical students and community agencies have expressed interest in having more hours in the community, either within this module or by collaboratively developing a more longitudinal module dedicated to community engagement and experiential learning. A continuous growth of this module can contribute to Community-College partnership and act as a channel to further support our College’s vision of social accountability, dedicated to “health equity, anti-racist education, community-based research, advocacy, authentic partnerships and the health needs of underserved and marginalized communities” ( College of Medicine, 2020).

There are limitations to our study. Our decision to gather data from two cohorts of medical students and community agency representatives in a rather limited time, was due to the time-sensitive objective and rather urgent need of evaluating and reforming the module. Longitudinal studies and tracking of students’ behaviour and perspectives could be more meaningful in understanding the long-term impact of this experimental learning module, particularly with regards to establishing mutual relationships between community and university. A two-survey approach (before and after module completion) could better evaluate the impact of the module but would likely suffer from responder fatigue and low response rates. It was already difficult for us to recruit participants for focus groups since medical students are notoriously busy with their studies. Future work might consider observational, perhaps ethnographic, approaches as a further, more detailed, form of data collection, in hopes of further enhancing students’ learning experiences about SDH and about the University’s collaboration with community partners. Even though this evaluation and learning module is specific to one institution, the model of teaching and learning through experiential learning is becoming universal across medical schools. Comparative studies among different medical schools would be beneficial to understand the impact of contextual and structural factors on students’ learning experiences in the communities, and contribute to the collective progress in teaching SDH and experiential learning in medical education.

## Conclusion

It is very worthwhile to evaluate CBE modules, such as our CWCLE module, together with students and community agencies. We feel gratified that we did not wait with our evaluation but acted as soon as we heard first misgivings about elements of the module, allowing us not only to improve this experiential learning module but also relations with community partners.

Our mixed method offered us a better understanding of module impact and opportunities for improvement. The quantitative part alone would not have brought up the issue of reciprocity; the qualitative part would not have shown us students’ differential preferences of the original module components. Also, it made an important difference to collaboratively redesign this CBE module with community agency representatives.

We recommend that, in future modifications of modules like this and in any learning opportunities that involve communities, medical schools should actively engage community agencies. Medical colleges should acknowledge these agencies for their expertise in community work and local challenges in SDH, offering them adequate compensation. Opportunities for community partners to influence decisions and recognition of their contributions to the training of medical students are forms of compensations beyond economic payments. Medical students’ engagement with such communities is crucial for their training and future practice. A mutual relationship between community agencies and medical colleges can benefit both medical students and community partners. It can sustain community-based programs that help students to become competent, socially accountable physicians who are better prepared to address the community needs and health disparities in society.

## Take Home Messages

• According to students and community agency representatives, medical students benefit from and appreciate community-based experiential learning of social determinants of health (SDH) and community resources.
• After completing a community-based experiential learning module, students reported an enhanced ability to perform all the module learning objectives and evidenced an impact on their attitudes towards SDH.
• Utilizing a mixed-method approach and collaborative framework, we demonstrate the benefits of developing community-based education opportunities to improve students’ learning experiences, promote community partnership, and achieve medical school’s mission of social accountability.

## Notes On Contributors

Yuchen Gao, BA, MA, is a Ph.D. candidate at the Department of Sociology, College of Arts and Science, University of Saskatchewan, Canada. Her thesis focuses on students’ learning of professionalism and professional identity formation in undergraduate medical education. ORCiD: https://orcid.org/0000-0001-6658-9928

Ulrich Teucher, paediatric nurse, PhD (Comp Lit), Associate Professor, Department of Psychology, College of Arts and Science. Associate member Department of Community Health and Epidemiology. University of Saskatchewan, Canada. He is a member of the Medicine and Society SoTL Cluster.

Erin Wolfson, MPA, MA, Community Engagement Specialist, Division of Social Accountability (DSA– https://medicine.usask.ca/social-accountability/index.php), College of Medicine, University of Saskatchewan, Canada. The DSA joins forces with communities to advance relevant and impactful health professional education, research, service, and advocacy through the lenses of equity, anti-oppression, authentic engagement, and excellence.

Krista Baerg, BSN, BA, MD, BScMed, FRCPC, is Associate Professor, Department of Pediatrics and Module Director, Patient and Family Centred Care Module, College of Medicine, University of Saskatchewan, Canada. She is a member of the Medicine and Society SoTL Cluster. ORCiD: https://orcid.org/0000-0001-5192-7285

Nicole Graham, Family Network & Youth Coordinator, Inclusion Saskatchewan, Saskatoon, Saskatchewan, Canada. Inclusion Saskatchewan ( https://www.inclusionsk.com/) is an agency that ensures citizens of Saskatchewan who have intellectual disabilities are valued, supported, and included members of society and have opportunities and choices in all aspects of life.

Sandra Pfeifer, BSW/RSW, Program Supervisor, Rainbow Youth Centre, Regina, Saskatchewan, Canada. The Rainbow Youth Centre ( http://www.rainbowyouth.com/) is a community-based, non-profit organization that aims to engage, educate, and inspire youth and families to lead healthy lives, providing education and support services for youth.

Krista Trinder, MA, CE, is the Program Evaluation Specialist at the Undergraduate Medical Education Office, College of Medicine, University of Saskatchewan, Canada. She chairs the Program Evaluation Sub-Committee and is responsible for ensuring that all required learning activities are evaluated.

Megan Wells, Manager of Inclusion, Inclusion Saskatchewan, Saskatoon, Saskatchewan, Canada. Inclusion Saskatchewan ( https://www.inclusionsk.com/) is an agency that ensures citizens of Saskatchewan who have intellectual disabilities are valued, supported, and included members of society and have opportunities and choices in all aspects of life.

Juan Nicolás Peña-Sánchez, MD, MPH, PhD, Assistant Professor, Department of Community Health and Epidemiology and Director CWCLE (Community and Workplace Centred Learning Experience) Module, College of Medicine, University of Saskatchewan, Canada. He is the project lead and co-principal investigator of the Medicine and Society SoTL Cluster. ORCiD: https://orcid.org/0000-0002-4653-525X
