# Supplementary material for: Evolution of a community-centred experiential learning module: A mixed-methods approach to promote social accountability and community partnership in undergraduate medical education
Source: MedEdPublish (2016). 2021 Jul 19;9:217. Originally published 2020 Oct 5. [Version 2] doi: 10.15694/mep.2020.000217.2 (PMC10699398; doi:10.15694/mep.2020.000217.2)
Supplement: Peer review history

# Reviewer report: Reviewer response for version 2

**DOI:** 10.21956/mep.20274.r31473

**Published:** 2021-09-03

**Authors:** Ken Masters

**Affiliations:** 1 Sultan Qaboos University

This review has been migrated. The reviewer awarded 5 stars out of 5 The authors have done a fine job of responding to the reviewers’ comments, and have improved their paper nicely. The addition of the extra supplementary files substantially increases the value of the paper for readers.Well done.

Reviewer Expertise:

NA

No decision status is available

# Reviewer report: Reviewer response for version 2

**DOI:** 10.21956/mep.20274.r31472

**Published:** 2021-08-02

**Authors:** Megan Anakin

**Affiliations:** 1 University of Otago

This review has been migrated. The reviewer awarded 4 stars out of 5 You have done a great job to address the review comments from both reviewers in this revised version of your article. The changes to your manuscript have enhanced the descriptions of your methods and results sections. The additional fine tuning of the aim has aligned it with the results. The discussion, conclusion, and take home messages sections are much stronger with the additions and changes. The new version of this article now reports the important lessons learned from your solid mixed-methods study to demonstrate the process your team used to improve an already successful community-centred experiential learning module.

Reviewer Expertise:

NA

No decision status is available

# Reviewer report: Reviewer response for version 1

**DOI:** 10.21956/mep.19007.r27270

**Published:** 2021-01-06

**Authors:** Ken Masters

**Affiliations:** 1 Sultan Qaboos University

This review has been migrated. The reviewer awarded 3 stars out of 5 An interesting article on the evolution of a community-centred experiential learning module. Overall, the paper is a good example of how to go about medication education research. The topic involves community-centred learning, and, right from the start, the community is involved in the process. Even in the original course, there was a good blend of community involvement, so this study demonstrates the process of improving something that is already good. The study itself is a solid mixed-methods study and reporting of quantitative and qualitative data, and the final model reflects the important lessons learned. (I’m not a great fan of using the cogs stock image, but that’s just a personal preference :-).As the first reviewer has pointed out, however, there are quite a few points that need to be addressed in the paper (I won’t repeat them here), so Version 2 of the paper will be a great improvement if they are addressed.Apart from the direct impact of the paper, I think an important sub-text is this: even if you have a good course, be vigilant for weaknesses, and when the rumblings grow, react by performing a detailed re-evaluation, as it will invariably lead to an even better outcome. Well done on that score.I look forward to Version 2 of the paper in which the issues are addressed.

Reviewer Expertise:

NA

No decision status is available

# Reviewer report: Reviewer response for version 1

**DOI:** 10.21956/mep.19007.r27271

**Published:** 2020-12-13

**Authors:** Megan Anakin

**Affiliations:** 1 University of Otago

This review has been migrated. The reviewer awarded 2 stars out of 5 Thank you for writing this article about your study because I am interested in how undergraduate medical education can be enhanced with experiential learning with communities. I have a few suggestions to enhance the presentation of this mixed methods study because it addresses four aims and presents a complicated set of results. Please consider rewording the first aim so that it more accurately reflects the data presented in the results section. The first aim does not appear to be addressed by the survey results presented in Table 1 because it reports self-evaluated ability rather than performance data about student achievement. In the methods section, please consider rephrasing the alpha level for the statistical tests performed to clarify that p-values less than .05 were considered significant. Please clarify if the same or a different set of interview questions was use for each group. Please consider putting the interview protocol used in an appendix. Please describe the steps used to analyse the data thematically so that researcher reflexivity is demonstrated. For example, please explain if the interview and focus group data from community representatives and students were analysed separately or combined and analysed together, and which authors were responsible for analysing this set of data. This additional detail will help to clarify the unit of analysis to the reader.In the results section, please present participant demographic of students (such as gender, age, ethnicity, entry to programme, relevant previous educational experiences) and for the 11 community representatives so the reader can evaluate the features of the sampled participants. In the results section, please consider revising how themes are presented so that the unit of analysis is clear to the reader. Please clarify that the themes represent the feedback the group of community representatives and students or as a comparison between the two groups. Please consider omitting the words “Some believed it…”, “Some students preferred…”, “Most students and agencies…” because when themes are reported they should be supported by representative extract to illustrate the description of each theme rather than to describe the feedback of only some members of the group. Please consider moving the last two paragraphs of the results to the discussion section since the ideas do not relate directly to the four aims of the study and the data collected. At the start of the discussion section, please consider providing a brief summary to revisit the aims of the study in light of the findings. This summary will help the reader better appreciate the implications and significance of the study’s main findings. To enhance the discussion of the limitations of the study, please consider the advice from: Lingard, L., 2015. The art of limitations. Perspectives on Medical Education, 4(3), pp.136-137.To enhance the second and third take home messages, please consider revising them to relate the points directly to the major findings of the study.I would be very happy to review a revised version of this article.

Reviewer Expertise:

NA

No decision status is available
